# Serological evidence of the circulation of the Rift Valley fever virus in sheep and goats slaughtered in Yaoundé, Cameroon

**DOI:** 10.1002/vms3.848

**Published:** 2022-05-20

**Authors:** Jean Thierry Ebogo‐Belobo, Serge Alain Sadeuh‐Mba, Georges Marc Arthur Mveng‐Sanding, Gwladys Monamele Chavely, Martin H. Groschup, Wilfred Fon Mbacham, Richard Njouom

**Affiliations:** ^1^ Department of Virology Centre Pasteur of Cameroon Yaoundé Cameroon; ^2^ Medical Research Centre Institute of Medical Research and Medicinal Plants Studies Yaoundé Cameroon; ^3^ Department of Biochemistry, Faculty of Science The University of Yaoundé I Yaoundé Cameroon; ^4^ Ministry of Livestock Fisheries and Animal Industries Yaoundé Cameroon; ^5^ Institute of Novel and Emerging Infectious Diseases Friedrich‐Loefer‐Institut Greifswald‐Insel Riems Germany

**Keywords:** Cameroon, livestock, Rift Valley fever virus, seroepidemiologic studies, slaughterhouse

## Abstract

**Background:**

Rift Valley fever (RVF) is an emerging mosquito‐borne haemorrhagic fever disease capable of causing severe outbreaks with high mortality and morbidity in human, livestock, and wildlife species, particularly in Africa. The onset of the disease in humans is often preceded by epizootic circulation in animals. Therefore, this study was conducted to investigate the seroprevalence of Rift Valley fever virus (RVFV) infection in animals slaughtered in the “Marché huitième” slaughterhouse in Yaoundé, Cameroon.

**Methods:**

A cross‐sectional study was conducted at the “Marché huitième” slaughterhouse in Yaoundé, Centre region of Cameroon in March 2020. Blood samples of two species of small ruminants (sheep and goat) were collected and processed. Serum was analysed for detection of RVFV IgG and IgM using commercial ELISA tests.

**Results:**

Of the 191 ruminants tested, RVFV IgG antibodies were positive in 10 (5.2%). Regarding categorization of the population based on the species and gender, sheep and female animal had the highest seroprevalence of 6.4% (3/47) and 7.0% (8/115), respectively. All sera from IgG antibodies‐positive samples were negative to IgM antibodies.

**Conclusion:**

This study provides evidence of the circulation of RVFV in small ruminants sold and slaughtered at the “Marché huitième” slaughterhouse in Yaoundé and highlights the need to develop a surveillance system for this virus encompassing humans, livestock, wildlife, and vectors in Cameroon.

## 1 INTRODUCTION

Rift Valley fever (RVF) is an emerging mosquito‐borne haemorrhagic fever disease causing severe outbreaks with high mortality and morbidity in human, livestock, and wildlife species, particularly in Africa (Linthicum et al., 2016; Nanyingi et al., 2015). It was first discovered in 1930 in Kenya (Daubney et al., 1931) and it is now endemic throughout multiple African countries and the Arabian Peninsula (Nanyingi et al., 2015). RVF is caused by Rift Valley fever virus (RVFV), which is an arbovirus in the *Phlebovirus* genus and Phenuiviridae family (Adams et al., 2017; King et al., 2018). The disease is transmitted by infected mosquitoes of several genera, mainly *Aedes* spp. and *Culex* spp., and by direct contact with body fluids, blood, or tissues of viremic animals (Balenghien et al., 2013; Bird et al., 2009; Pepin et al., 2010).

RVFV infections in humans usually lead to subclinical infection or cause moderate to severe non‐fatal, acute illness but some patients can develop haemorrhagic syndrome and/or encephalitis (Pepin et al., 2010) with an overall case fatality rate estimated to be between 0.5% and 2% (Madani et al., 2003; Pepin et al., 2010). In animals, infections are characterized by abortion storms, especially among pregnant domestic ruminants like goats and sheep and high mortality among newborns of domestic ruminants (Bird et al., 2009).

An increase in international domestic ruminants trade and some other factors encountered in Sub‐Saharan Africa such as favourable climate, presence of a wide variety of vectors, and human behaviour may lead to RVFV outbreak (Baba et al., 2016), which can have considerable socio‐economic and public health impacts (Baba et al., 2016; Peyre et al., 2015). The onset of the disease in humans is preceded generally by epizootic circulation of the virus in livestock. Surveillance of RVF by assessing past and present RVFV antibodies status in animals and humans may be a good indicator providing evidence of the circulation of the virus in a given area and helps to detect at‐risk populations, to anticipate a potential outbreak.

In Cameroon, there is not yet any official report of RVF outbreaks but the circulation of the virus has been confirmed by reports of detection of antibodies against the virus in animals and humans (Gonzalez et al., 1989; LeBreton et al., 2006; Poueme et al., 2019; Rissmann et al., 2017; Sadeuh‐Mba et al., 2018; Zeller et al., 1995). Of these previous studies on RVFV carried out in Cameroon on animals, only two studies were carried out in the city of Yaoundé and the samples of small ruminants collected were less than 15 (LeBreton et al., 2006; Rissmann et al., 2017). This silent circulation of RVFV shows that Cameroon may be a country at risk of RVF epidemics and indicates the need of continuous surveillance of this virus in both animals and humans. This study investigates the seroprevalence of RVFV infection in animals slaughtered in “Marché huitième” slaughterhouse, the main slaughter market for small ruminants in Yaoundé, Cameroon.

## 2 MATERIALS AND METHODS

### 2.1 Study area

**FIGURE 1:**
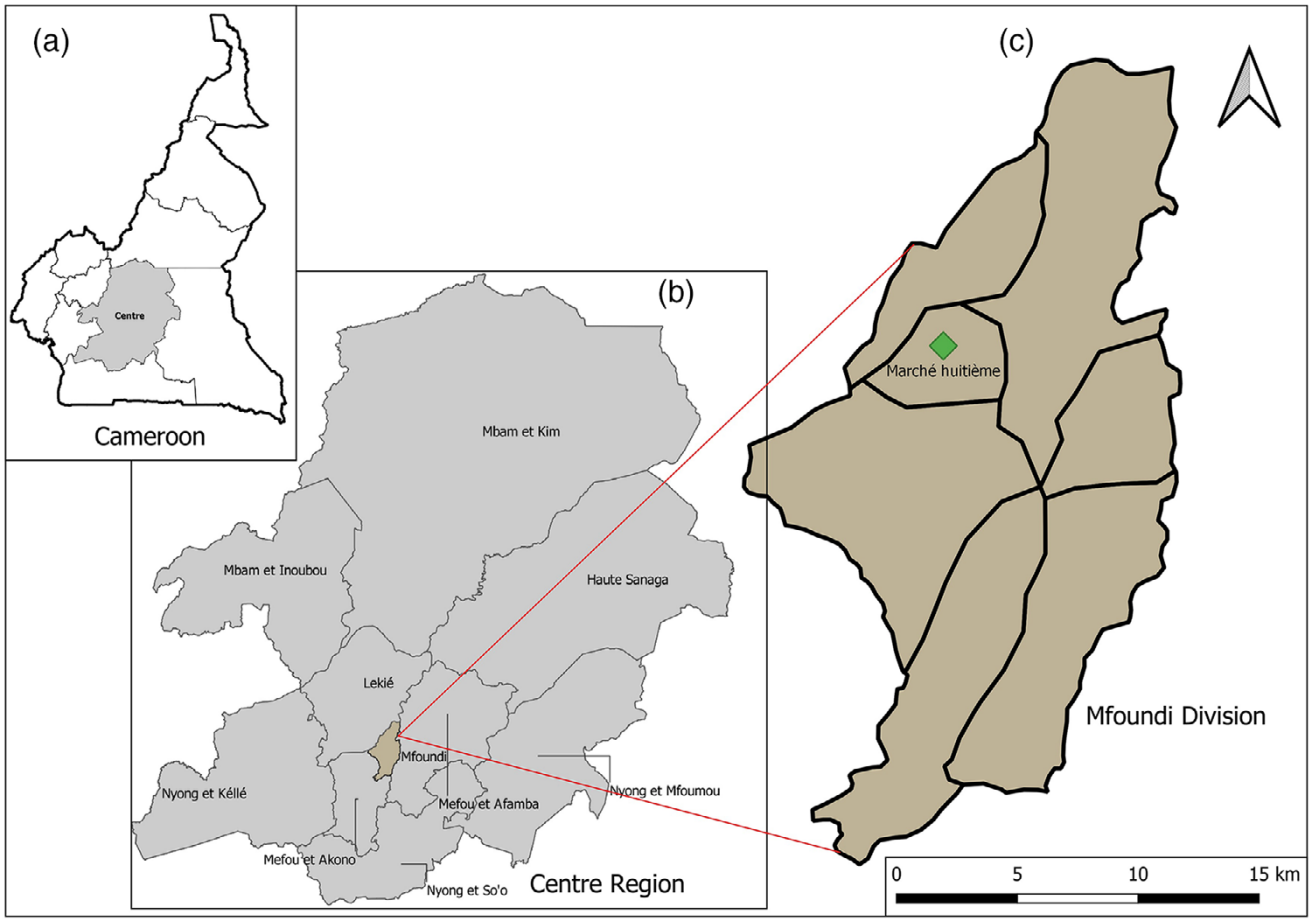
Geographical representation of study area. Panel (a) is highlighting the Centre region, panel (b) is highlighting the Mfoundi division, and panel (c) is highlighting the sampling points in green.

This study was carried out at the “Marché huitième” slaughterhouse in Yaoundé, Mfoundi division, Centre region of Cameroon (Figure 1) between 4 and 20 March 2020, at the end of dry season. This market is the largest for the sale and slaughter of small ruminants in Yaoundé. An average of 150 domestic ruminants are slaughtered per day in this slaughterhouse for human consumption in private homes or delivered to supermarkets in the city (the city of Yaoundé has over 2.766 million inhabitants) and its surroundings for sale. The small ruminants slaughtered in this slaughterhouse are from various sources, mainly from the northern part of the country.

### 2.2 Blood sampling and laboratory analysis

A blood sample (5 ml) was collected consecutively from 191 small ruminants, including 47 sheep and 144 goats, through puncture of the jugular vein before the animal was slaughtered according to good veterinary practices. The blood samples were carried in an icebox with ice packs to the virology laboratory of the Centre Pasteur du Cameroun (CPC). Serum was later separated from the whole blood and stored at –80°C until tested.

For antibody screening, sera were analysed using the commercially available competitive ELISA kit from Innovative Diagnostics (IDvet^®^ ID Screen Rift Valley Fever Competition Multi‐species, Grabels, France) for the detection of both IgG and IgM antibodies according to the manufacturer's instruction. Results’ interpretation was determined by calculating the inhibition percentage (S/N %). All samples giving a percentage of inhibition higher than 50% were defined as negative and those with a percentage of inhibition lower than 40% as positive. Samples giving a percentage of inhibition between 40% and 50% were considered inconclusive.

All samples testing positive to the Competition‐ELISA were re‐analysed using the IgM capture ELISA (IDvet^®^ ID Screen Rift Valley Fever IgM Capture, Grabels, France) according to the manufacturer's instructions for specific detection of IgM antibodies. All samples with a percentage of inhibition higher than 50% were considered to be positive and those lower than 40% as negative. Sample with a percentage of inhibition between 40% and 50% were identified as inconclusive. All samples that tested positive for total RVFV antibodies but negative for IgM were considered as anti‐RVF IgG antibodies positive.

### 2.3 Data analysis

The data were entered into a Microsoft Excel spreadsheet and imported into Statistical Package for Social Sciences (SPSS) software Version 23.0. Seroprevalence for RVFV was analysed by species and sex, presented as proportions, and compared using Fisher's exact test. The differences were considered significant where the P‐values were lower than 0.05.

## 3 RESULTS

The overall seroprevalence was 5.2% (10/191) for anti‐RVFV IgG antibodies and 0.0% (0/191) for IgM antibodies. The seroprevalence for anti‐RVFV IgG antibodies was 6.4% (3/47) in sheep and 4.9% (7/144) in goats. Eight of the 10 positive sera were from female livestock (7.0%). Two inconclusive sera were observed and were from female livestock (Table 1).

**TABLE 1:** Seroprevalence of RVFV IgG antibodies in small ruminants slaughtered at “Marché huitième” slaughterhouse

	No of sample	Positive	Negative	Inconclusive	Seroprevalence (%)	*p*‐value
Species						
Sheep	47	3	43	1	6.4	0.642
Goat	144	7	136	1	4.9	
Sex						
Female	115	8	105	2	7.0	0.208
Male	76	2	74	0	2.6	
Total	191	10	179	2	5.2	

## 4 DISCUSSION

Here, we report the presence of IgG antibodies against RVFV in sheep and goats slaughtered at the “Marché huitième” slaughterhouse of Yaoundé. The overall seroprevalence of RVFV IgG antibodies, which is indicative of past exposure, was 5.2%, and none of these small ruminants showed evidence of new or recent infection. The presence of IgG antibodies against RVFV among small ruminants provides evidence of the silent circulation of RVFV in Cameroon and indicates that humans, particularly breeders, workers in slaughterhouses, and livestock markets are exposed to RVFV. This silent circulation in animals, reported by previous studies in Cameroon, confirms previous findings (Gonzalez et al., 1989; LeBreton et al., 2006; Poueme et al., 2019; Rissmann et al., 2017; Sadeuh‐Mba et al., 2018; Zeller et al., 1995).

The prevalence of IgG antibodies against RVFV for small ruminants found in this study is slightly higher compared to those found in other recent studies in Cameroon (Poueme et al., 2019; Rissmann et al., 2017). However, when we consider climatic/seasonal conditions (dry season) and locality (Centre region), the results of this study are similar to those obtained in previous studies (Poueme et al., 2019; Rissmann et al., 2017), suggesting the influence of climatic/seasonal and agroecological conditions in the variation of seroprevalence.

Cameroonian borders in the northern part of country are sufficiently porous and unrestricted for the movement of humans and animals and some animals sold and slaughtered at the “Marché huitième” are likely to come from these neighbouring countries. Serological and molecular studies carried out in neighbouring countries that share common borders showed a variable seroprevalence of prior and current/recent infections (Nakouné et al., 2016; Opayele et al., 2019; Ringot et al., 2004). These findings indicate possible transboundary silent circulation of RVFV between Cameroon and its neighbouring countries such as Nigeria, Chad, and the Central African Republic.

Among small ruminants, sheep had the highest seroprevalence in this study (6.4%). This result is consistent with previous work (Budasha et al., 2018; Nakouné et al., 2016; Poueme et al., 2019; Ringot et al., 2004), suggesting that sheep must be a species preferentially infected with RVFV among small ruminants. Moreover, sheep are mainly reared in the northern region of the country (Ministry of Livestock, Fisheries and Animal Industries, n.d.; Poueme et al., 2019) and its movement from the Northern to other regions of the country may be a possible promoting factor for sporadic transmission and circulation of RVFV in Cameroon.

## 5 CONCLUSION

In summary, the findings of this study provide evidence that RVFV circulates in small ruminants sold in “Marché huitième” and suggest that breeders, workers in slaughterhouses, or livestock markets are likely to be exposed to RVFV infections, even without reports of clinical signs in susceptible hosts in Cameroon. Therefore, this study emphasizes the need to develop adequate control measures to track their geographic spread and limit the RVFV circulation and transmission to animals and humans in Cameroon. This surveillance should focus on those involved in the trade and breeding of small ruminants to estimate the actual exposure of the virus in humans.

## CONFLICT OF INTEREST

The authors declare no conflict of interest.

## ETHICS

The authors confirm that the ethical policies of the journal, as noted on the journal's author guidelines page, have been adhered to. Legal permission for collect samples from small ruminants was obtained from the Regional Delegation of Livestock, Fisheries and Aminal Industries of Centre Region/Cameroon (No. 000021/L/MINEPIA/SG/DREPIA‐CE, Date: 18 February 2020).

## AUTHOR CONTRIBUTIONS

J.T.E.‐B. performed investigation and formal analysis and wrote the original draft. S.A.S.‐M. provided resources. S.A.S.‐M., M.H.G., W.F.M., and R.N. designed methodology. S.A.S.‐M., G.M.A.M.‐S., G.M.C., M.H.G., W.F.M., and R.N. reviewed and edited the manuscript. M.H.G., W.F.M., and R.N. conceptualized the idea of the study. R.N. performed validation and supervision.

### PEER REVIEW

The peer review history for this article is available at https://publons.com/publon/10.1002/vms3.848.

## Data Availability

The data that support the findings of this study are available from the corresponding author upon reasonable request.
